# Eco-Virological Approach for Assessing the Role of Wild Birds in the Spread of Avian Influenza H5N1 along the Central Asian Flyway

**DOI:** 10.1371/journal.pone.0030636

**Published:** 2012-02-07

**Authors:** Scott H. Newman, Nichola J. Hill, Kyle A. Spragens, Daniel Janies, Igor O. Voronkin, Diann J. Prosser, Baoping Yan, Fumin Lei, Nyambayar Batbayar, Tseveenmyadag Natsagdorj, Charles M. Bishop, Patrick J. Butler, Martin Wikelski, Sivananinthaperumal Balachandran, Taej Mundkur, David C. Douglas, John Y. Takekawa

**Affiliations:** 1 EMPRES Wildlife Unit, Emergency Centre for Transboundary Animal Diseases, Animal Production and Health Division, Food and Agriculture Organization of the United Nations, Rome, Italy; 2 United States Geological Survey, Western Ecological Research Center, San Francisco Bay Estuary Field Station, Vallejo, California, United States of America; 3 Wildlife Health Center, School of Veterinary Medicine, University of California, Davis, California, United States of America; 4 Department of Biomedical Informatics, The Ohio State University, Columbus, Ohio, United States of America; 5 United States Geological Survey, Patuxent Wildlife Research Center, Beltsville Lab, Beltsville, Maryland, United States of America; 6 Computer Network Information Center, Chinese Academy of Sciences, Beijing, China; 7 Institute of Zoology, Chinese Academy of Sciences, Beijing, China; 8 Wildlife Science and Conservation Center, Bayanzurkh District Ulaanbaatar, Mongolia; 9 Ornithological Laboratory, Institute of Biology, Mongolian Academy of Sciences, Ulaanbaatar, Mongolia; 10 Bangor University, School of Biological Sciences, Brambell Laboratories, Bangor, Gwynedd, United Kingdom; 11 School of Biosciences, University of Birmingham, Birmingham, United Kingdom; 12 Max Planck Institute for Ornithology, Radolfzell, Germany; 13 Bombay Natural History Society, Hornbill House, Mumbai, India; 14 Wetlands International, 471, 6700 AL, Wageningen, The Netherlands; 15 United States Geological Survey, Alaska Science Center, Juneau, Alaska, United States of America; University of Durham, United Kingdom

## Abstract

A unique pattern of highly pathogenic avian influenza (HPAI) H5N1 outbreaks has emerged along the Central Asia Flyway, where infection of wild birds has been reported with steady frequency since 2005. We assessed the potential for two hosts of HPAI H5N1, the bar-headed goose (*Anser indicus*) and ruddy shelduck (*Tadorna tadorna*), to act as agents for virus dispersal along this ‘thoroughfare’. We used an eco-virological approach to compare the migration of 141 birds marked with GPS satellite transmitters during 2005–2010 with: 1) the spatio-temporal patterns of poultry and wild bird outbreaks of HPAI H5N1, and 2) the trajectory of the virus in the outbreak region based on phylogeographic mapping. We found that biweekly utilization distributions (UDs) for 19.2% of bar-headed geese and 46.2% of ruddy shelduck were significantly associated with outbreaks. Ruddy shelduck showed highest correlation with poultry outbreaks owing to their wintering distribution in South Asia, where there is considerable opportunity for HPAI H5N1 spillover from poultry. Both species showed correlation with wild bird outbreaks during the spring migration, suggesting they may be involved in the northward movement of the virus. However, phylogeographic mapping of HPAI H5N1 clades 2.2 and 2.3 did not support dissemination of the virus in a northern direction along the migration corridor. In particular, two subclades (2.2.1 and 2.3.2) moved in a strictly southern direction in contrast to our spatio-temporal analysis of bird migration. Our attempt to reconcile the disciplines of wild bird ecology and HPAI H5N1 virology highlights prospects offered by both approaches as well as their limitations.

## Introduction

Avian influenza virus of the highly pathogenic subtype H5N1 (or ‘HPAI H5N1’), continues to pose a pandemic threat more than a decade after the virus first emerged in 1996 [1]. The virus has not yet gained capacity for rapid human-to-human transmission but shows high fatality rates in humans (60%) [2]. It has become endemic in Indonesia, Bangladesh, India and Egypt with repeated emergence in China, Vietnam, Thailand and Mongolia [3]. HPAI H5N1 also remains a significant threat to the poultry industry, destabilizing agriculture in countries where backyard farming of domestic ducks is common [4] and impacting the food security and livelihood of millions of people. Free-ranging domestic ducks have been implicated as the reservoir of HPAI H5N1 in South and Southeast Asia [5], [6], [7] where domestic ducks forage on post-harvested rice fields [8], [9]. In the laboratory, ducks may experience productive infections with HPAI H5N1 in the absence of clinical symptoms and transmit virus to susceptible individuals [10], [11]. However, movement of HPAI H5N1 over long distances has been attributed to migratory wild birds [12], [13], [14] as well as marketing of poultry [15], [16] and illegal trade of wild birds [17]. Wild birds may act as temporary vectors for HPAI H5N1 as suggested by the large-scale outbreak at Qinghai Lake in 2005 [18], [19], extrapolation from exposure trials of captive birds [14], [20], [21] and inference from introduction of HPAI H5N1 into Europe on several occasions [22]. However, the role of wild and domestic birds in the transmission of HPAI H5N1 depends on temporal and regional contexts and is currently far from clear [16].

Since 2005, wild bird mortalities resulting from infections of HPAI H5N1 have been reported from 38 countries in Asia, Europe, Africa and the Middle East [23]. A unique pattern of infection has emerged during the spring migration of wild birds through East Asia including China, Mongolia and Siberia [3]. Outbreaks of HPAI H5N1 in wild birds in this region have been reported in 2005, 2006, 2009 and 2010, commencing between March and April [3] (see Fig. S1). This pattern suggests outbreaks in this region are initiated by the northward migration of wild birds commencing in the spring. The region of East Asia where outbreaks have occurred supports few poultry but is a major migration corridor and breeding area for waterfowl in the Central Asian Flyway, an area extending from India, Bangladesh and Myanmar in the south to Siberia in the north. Recently, a two-host model has been proposed to account for the recurring HPAI H5N1 outbreaks in this region [24] whereby: 1) domestic ducks act as reservoirs for the virus in South and Southeast Asia; 2) interaction between domestic and wild birds occurs during outbreaks of HPAI H5N1 at the southern end of the Central Asian Flyway and 3) wild birds move the virus northwards into East Asia during spring migration. To test this model, further information is needed on the spatio-temporal correlation of wild bird migration and virus movement.

The bar-headed goose (*Anser indicus*) and ruddy shelduck (*Tadorna ferruginea*) are among the primary hosts reported with HPAI H5N1 infections during wild bird outbreaks in the Central Asian Flyway [3]. The bar-headed goose was the primary species affected at the Qinghai Lake outbreak in 2005 [18], [25] and their migration has been correlated with the introduction of the virus from the poultry-intensive area of Lhasa in Tibet [26]. Moreover, while many bird species die shortly after HPAI H5N1 exposure in the lab, the bar-headed goose is an exception and can remain asymptomatic for 6.5 days and survive infection resulting from contact with clinically infected birds [20]. The ruddy shelduck is a less robust host showing 100% mortality in response to infection from ‘contact’ birds; however this species can remain asymptomatic for 5.5 days after exposure in a laboratory environment [27]. Satellite telemetry data has recently been used to determine how far these species can travel within the asymptomatic period defined by laboratory studies [14]. A more comprehensive approach to understanding waterfowl-mediated dispersal of HPAI H5N1 involves accounting for variation in migration strategies shown within a species. A detailed investigation of the migration ecology of the bar-headed goose and ruddy shelduck is therefore needed to provide a more accurate estimation of their dispersal potential between wintering, stopover and breeding sites that punctuate movement along a flyway.

As a step towards assessing the role of wild birds in the dispersal of HPAI H5N1 from South to East Asia, we developed an eco-virological approach that utilized tools from the disparate fields of ecology and virology. We compared the migration of waterfowl populations marked with highly-accurate GPS satellite transmitters during 2005–2010 to both: 1) the spatio-temporal patterns of poultry and wild bird outbreaks of HPAI H5N1, and 2) the trajectory of the virus in the outbreak region based on phylogeographic mapping. Our specific objectives were: i) to test for spatial and temporal correlation of outbreaks with the northward movement of birds during spring migration, and ii) to assess whether novel strains of HPAI H5N1 originating from parental strains were found in more northern areas as migration progressed. Unlike risk-mapping or phylogenetic studies conducted at the regional [28] or global scale [13], [29], [30], we investigated virus movement at a scale relevant to the wild bird hosts, the Central Asian Flyway. This allowed us to account for distribution patterns of individual host species, thereby recognizing the importance of interspecific differences in migration ecology that influence wild bird-mediated dispersal of HPAI H5N1. Ultimately, the combined use of ecologic and virologic tools represents one of the first attempts to simultaneously characterize the movement of host and virus within a potential thoroughfare for HPAI H5N1.

## Materials and Methods

### Capture sites

Capture of bar-headed geese and ruddy shelduck took place in four countries within the Central Asian Flyway: India (Keoladeo National Park), Nepal (Chitwan National Park), China (Qinghai Lake National Nature Reserve) and Mongolia (Khorgo-Terkhiin Tsagaan Nuur National Park). Keoladeo National Park, India (27°9′N, 77°30′E) is west of Bharatpur in the state of Rajasthan [31]. The 30 km^2^ park was originally developed as a waterbird area for hunting in 1899 by the Maharaja of Bharatpur and supports a high abundance and diversity of migratory and resident waterbirds [32]. A manmade wetland in the floodplain of the Gambhir and Banganga River, inundation occurs during October, drops through the winter and then quickly dries from March through June [33]. Chitwan National Park (27°30′N, 84°30′E) is the oldest national park in Nepal, located 10 km west of Bharatpur (Narayangadh) in the sub-tropical Terai lowlands of South-central Nepal. The park is 930 km^2^ and is bisected by floodplains of the Narayani River and provides habitat for more than 450 species of birds [34]. Qinghai Lake National Nature Reserve, China (36°49′N, 99°49′E) is located in the northeastern Qinghai-Tibet Plateau, 280 km west of Xining in Qinghai Province [35]. Qinghai Lake sits at an elevation of 3,200 m and is the largest saltwater lake in China with an area of 530 km^2^. It is a migration bottleneck for waterbirds in the Central Asian Flyway, where many species converge to stop-over, molt or breed. Three small island complexes within the lake constitute breeding areas for bar-headed geese, brown-headed gulls (*Larus brunnicephalus*), black-headed or Pallas's gulls (*Larus ichthyaetus*) and great cormorants (*Phalacrocorax carbo*). Khorgo-Terkhiin Tsagaan Nuur National Park (48°8′N, 99°38′E) is 773 km^2^ and situated at an altitude of 2060 m in the Khangai Mountains of Taryat Soum in Arkhangai Aimag, central Mongolia. The park encompasses Terkhiin Tsagaan Lake (or ‘White Lake’), a freshwater body 268 km^2^ in size that provides breeding habitat for migratory waterfowl.

### Capture and marking with satellite transmitters

Capture occurred in India during February 2005, December 2008 and 2009; Nepal in February 2005; China in March and September 2007 and 2008; and Mongolia during July 2008 and 2009. Birds were captured with leg nooses consisting of monofilament loops attached to wooden sticks connected with nylon cord in lines of 50–100 nooses, or they were captured during their flightless molting period by herding them into drive-traps. Upon capture, birds were immediately placed in individual cloth bags or held in corrals and processed to record sex, age, weight, mass, culmen, flat wing, and diagonal tarsus. Birds were marked with 30 g or 45 g GPS solar-powered platform terminal transmitters or ‘PTTs’ (Microwave Telemetry, Inc., Columbia, MD, USA). PTTs were secured to birds with a Teflon harness (Bally Ribbon Mills, Bally, PA, USA). Transmitter packages averaged <3% of a bird's body mass. Birds were released near capture locations as soon as possible after processing (2–12 h). GPS PTTs were programmed to record 6–12 GPS locations each day. Transmissions relaying the GPS locations were received by the Argos satellite tracking system (CLS America Inc., Largo, MD, USA). Tracking data through July 2010 were included in this study. We used ArcGIS 9.3 (Environmental Systems Research Institute, Inc., Redlands, California, USA) and Google Earth 5.0 (Google, Mountain View, California, USA) to visualize telemetry locations.

### Ethics statement

This study was carried out in strict accordance with the recommendations of the Ornithological Council ‘Guidelines to the Use of Wild Birds in Research’. Capture permits were obtained from the relevant government authority in India, China and Mongolia. Procedures for capture, handling, and marking were approved by a U.S. Geological Survey Animal Care and Use Committee and the University of Maryland Baltimore County Institutional ACUC (Protocol EE070200710).

### HPAI H5N1 outbreak data

Information about HPAI H5N1 outbreaks (confirmed only) were obtained from the FAO EMPRES-i Database [3]. Outbreaks were defined as mass-mortality events. EMPRES-i collects outbreak information from official sources (e.g. World Health Organization, Office International des Epizooties and the European Union), unofficial sources (e.g. country project reports or field mission reports) and disseminated reports (e.g. the Program for Monitoring Emerging Infectious Diseases – ProMed). Outbreaks are carefully checked before being entered into the database [36]. Metadata associated with each outbreak includes: date, location (country, administrative region, locality, latitude and longitude), and whether the outbreak occurred in domestic or wild birds. Outbreak data was selected for the period January 2005 to July 2010, coinciding with the satellite tracking of wild birds.

### Spatio-temporal analysis of wild bird movements and HPAI H5N1 outbreaks

Telemetry data from 2005–2010 were combined to present a generalized pattern of wild bird movements. For birds tracked over multiple years, each year of data was treated as an independent observation. The spatial and temporal extent of wild bird movements were characterized by constructing utilization distributions (UDs) that accounted for latitude, longitude and time [37]. Three dimensional UDs are unique in capturing temporal variations in the probability of occurrence, and are ideal for modeling the time-sensitive movements of migratory species. Each UD spanned a 14-day period; the sum of the asymptomatic period in bar-headed geese, 5–8 days [20] and ruddy shelduck 6 days [27] and estimated time taken to detect the outbreak in East Asia (7 days). The UDs were generated on a constant 125 km resolution spatial grid and a 125 km smoothing parameter. The UDs were contoured at the 99% level to generate the final polygons of wild bird distributions for co-analysis with the virus outbreak polygons.

To determine the spatial extent of outbreaks we assessed which poultry and wild bird outbreaks fell within the migration corridor of bar-headed geese and ruddy shelducks. This delimited outbreaks within a feasible distance for wild birds to reach. Migration corridors for each species were determined using, a single, daily location for each bird to generate minimum convex polygons (MCP) with Animal Movement Extension [38]. Unlike the biweekly UDs, the MCP was not partitioned according to time. The MCPs were overlaid with outbreaks (from EMPRES-i database) to define cases to be included in subsequent analysis (Fig. S2).

The biweekly UDs were spatially intersected with the outbreaks that occurred within each 14-day period, but temporally offset so that UDs preceeded outbreaks by 7 days. This approach assumed a time lag between the arrival of a bird and the start of an outbreak owing to exposure, incubation, and the onset of symptoms. We counted the number of outbreaks that 1) fell inside the biweekly UD and 2) fell outside the UD but inside the MCP boundary that delimited outbreaks potentially caused by a wild bird. Fisher Exact tests were used to determine if the proportion of outbreaks ‘inside’ differed significantly from ‘outside’ compared with expected values derived from the area of the UD. Tests were performed for each 14-day period, with poultry and wild bird outbreaks treated separately. A Bonferroni adjustment for multiple tests was not applied since our null hypothesis of interest was not tested simultaneously, but over discrete biweekly periods throughout the wild bird annual cycle. Also, erroneous application of Bonferroni adjustments would risk an increase in Type II error (false negative) [39].

### Phylogeographic mapping of HPAI H5N1

Phylogeographic mapping of HPAI H5N1 viruses that overlapped with the Central Asian Flyway was performed to qualitatively assess the trajectory of the virus compared to the northward migration of bar-headed geese and ruddy shelduck. We chose to include sequences from poultry and wild birds as transmission of the virus is thought to involve exchange between the two avian populations. Hemagglutinin (HA) nucleotide sequences for low and highly pathogenic H5N1 influenza were obtained from the Genbank database hosted by the National Institutes of Health (http://www.ncbi.nlm.nih.gov) and the Global Initiative on Sharing of All Influenza Data (http://gisaid.org). Metadata associated with each sequence provided the geographic location for the outbreak. Where possible we attempted to classify each site at the provincial or state level. We aligned H5N1 (HA) nucleotide data using the software package ClustalW-MPI [40]. Sequence ends were trimmed to the reading frame. We removed sequences that broke the protein reading frame. The final alignment consisted of 3304 sequences and 1776 aligned positions. A phylogeny was obtained via tree search of 100 replicates using the maximum likelihood (ML) criterion implemented in RAxML under the GAMMAI model of nucleotide substitution [41]. The tree was rooted to A/goose/Guangdong/1/96 and included sequences from January 1959 (A/chicken/Scotland/1959) to June 2010 (A/Hubei/1/2010). We used character optimization in Tree Analysis Using New Technology (or ‘TNT’) [42] to identify clades of HPAI H5N1 that circulate in the Central Asian Flyway. Sequences within each clade were realigned and new proximal outgroups were chosen. Transmission events were calculated using a modified version of the method developed by Slatkin and Maddison [43]. To find transmission events, different geographical regions were treated as character states of a single multistate character, and were mapped onto trees obtained from phylogenetic analyses using standard optimization methods. To assess directionality of strain evolution, the change command in TNT was used to count the minimum and maximum number of transmission events for pairs of geographical character states for all global parsimonious reconstructions of the multistate character. When the number of state changes for a geographical state pair was >0, this result was interpreted as a possible transmission route from one region to another. Patterns of viral evolution were not assessed by migration season owing to the small number of sequences available for wild birds in the Central Asian Flyway. We visualized these transmission routes and their polarity with ROUTEMAP, http://routemap.osu.edu [44].

## Results

### Migration ecology

In total, 97 bar-headed geese were marked with GPS satellite transmitters; 28 from India, 1 from Nepal, 29 from China and 39 from Mongolia. Telemetry data were filtered to obtain a single location per day (to avoid serial autocorrelation between locations within 24 h). GPS locations were obtained from February 2005 to July 2010 for an average of 348 days, (range: 9–1216 days) or 137 locations per bird (range: 4–501 locations). Of the 97 marked bar-headed geese, 69 completed one or more migratory seasons and were included in statistical analysis.

**Figure 1:**
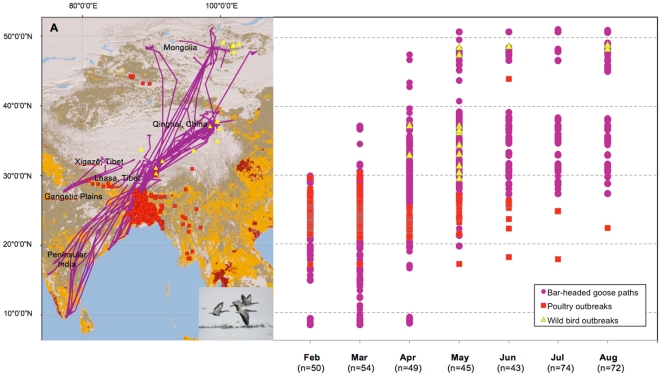
Northward movement of bar-headed geese in relation to HPAI H5N1 outbreaks in the Central Asian Flyway, 2005–2010. Paths of bar-headed geese are indicated in purple, with outbreaks from poultry (red squares) and wild birds (yellow triangles). Poultry density (individuals/km^2^) in the Central Asian Flyway ranges from high (10,000–100,000: dark red), medium (1,000–10,000: orange), low (1–1,000: brown) and absent (grey).

The migration of marked bar-headed geese followed the Central Asian Flyway (Fig. 1); however, there was variation among their migratory distance and duration. Bar-headed geese followed five different spring migration routes; a) ‘Gangetic Plain’ (lowlands of Northern India and Southern Nepal: wintering) to the Xigazê Prefecture, Tibet (breeding) completed in 22 days (n = 4, S.D. = 20.4), b) Peninsular India to Lhasa Prefecture, Tibet completed in 60 days (n = 3, S.D. = 18.0), c) Lhasa Prefecture to Qinghai Lake completed in 28 days (n = 9, S.D. = 15.0), d) Peninsular India to Qinghai Lake completed in 60 days (n = 4, S.D. = 12.1), and e) Peninsular India to Mongolia completed in 68 days (n = 5, S.D. = 17.9) (Table S1a). The annual cycle for all geese was categorized as follows: wintering (25 Nov–17 Mar), spring migration (18 Mar–9 Jun), breeding (10 Jun–1 Sep) and autumn migration (2 Sep–24 Nov).

A total of 44 ruddy shelduck were marked with GPS satellite transmitters; 8 from India and 36 from China. GPS locations were obtained from April 2007 to July 2010 for an average of 356 days (range: 11–1041 days). As above, data were filtered to obtain a single daily location, resulting in an average of 267 locations per bird (range: 11–727 locations). Of the 44 marked ruddy shelduck, 33 (75%) contributed daily locations for statistical analysis.

**Figure 2:**
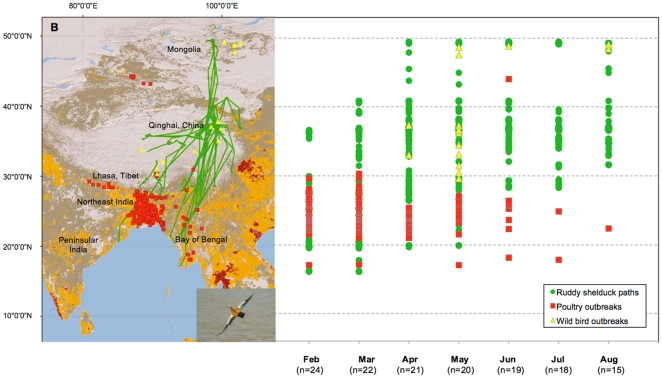
Northward movement of ruddy shelduck in relation to HPAI H5N1 outbreaks in the Central Asian Flyway, 2005–2010. Paths of ruddy shelduck indicated in green, with outbreaks from poultry (red squares) and wild birds (yellow triangles). Poultry density (individuals/km^2^) in the Central Asian Flyway ranges from high (10,000–100,000: dark red), medium (1,000–10,000: orange), low (1–1,000: brown) and absent (grey).

The migration routes of all ruddy shelduck conformed to the eastern boundary of the Central Asian Flyway (Fig. 2), also considered the western part of the East Asian Flyway under some flyway designations [45]. Ruddy shelduck followed four different spring migration routes; a) Northeast India (wintering) to Lhasa Prefecture, Tibet (breeding) completed in 39 days (n = 1), b) Peninsular India to the Nagqu Prefecture, Tibet, completed in 66 days (n = 1), c) the ‘Bay of Bengal’ (surrounding coasts of India, Bangladesh and Myanmar) to Qinghai Lake, completed in 10 days (n = 18, S.D. = 10.6), and d) Bay of Bengal to Mongolia completed in 11 days (n = 4, S.D. = 6.2) (Table S1b). Stages within its annual cycle were categorized as follows: wintering (25 Nov–3 Mar), spring migration (4 Mar–9 Jun), breeding (10 Jun–18 Aug) and autumn migration (19 Aug–24 Nov).

### Potential for dispersal of HPAI H5N1 during the spring migration

The ‘dispersal potential’ or the likelihood that bar-headed geese performed long-distance movements within the approximately 7-day asymptomatic period varied among the five migration routes (Table S1a). Total migration distance for each individual was defined by the Euclidian distance between its northernmost and southernmost location. Geese migrating from Peninsular India to Mongolia showed the greatest potential to move within an infection-relevant time frame from India to Lhasa (0.114), Lhasa to Qinghai (0.055) and Qinghai to Mongolia (0.282). Geese that migrated from Peninsular India to Qinghai had a 0.019 probability of moving 1,200 km between India and Lhasa, within an infection-relevant time frame. Geese with wintering and breeding sites relatively close together (i.e. Peninsular India to Lhasa, Gangetic Plain to the Tibetan Plateau and Lhasa to Qinghai) did not complete migration within the asymptomatic period.

The dispersal potential was higher for ruddy shelduck migrating from the Bay of Bengal to Mongolia than for the Bay of Bengal to Qinghai Lake (Table S1b). Ruddy shelduck migrating to Mongolia had a 0.693 likelihood of moving 1,200 km within an infection-relevant time frame between the Bay of Bengal and Qinghai Lake, and 0.708 probability between Qinghai Lake and Mongolia. Ruddy shelduck migrating a shorter distance to breeding grounds in Qinghai Lake had a 0.256 likelihood of moving 1,200 km between the Bay of Bengal and Qinghai Lake within an infection-relevant time. Ruddy shelduck migrating over the relatively short distance from Peninsular or northeast India to breeding sites in the Tibetan Plateau did not complete more than a 500 km flight within the 7-day asymptomatic period.

### Wild bird annual cycle in relation to HPAI H5N1 outbreaks

**Figure 3:**
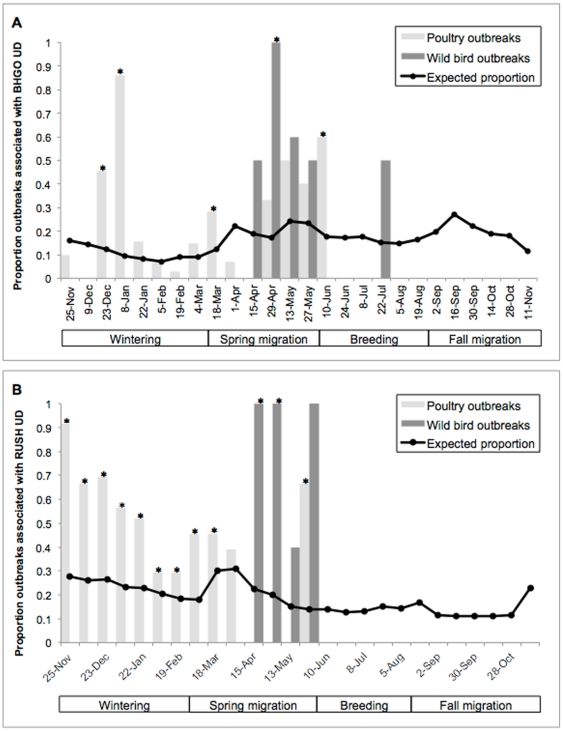
Association of HPAI H5N1 outbreaks with wild bird utilization distributions (UD) during the annual cycle, 2005–2010. The proportion of outbreaks (grey columns) associated with (a) bar-headed geese and (b) ruddy shelduck UDs is compared to expected values (black line). Significant associations (p≤0.05) are indicated by an asterisk (*).

We generated 26 biweekly UDs from the annual movement of bar-headed geese and ruddy shelduck (Table 1). For the bar-headed goose, 19.2% (5/26) of their UDs were significantly associated with HPAI H5N1 outbreaks (Fig. 3a). Association with poultry outbreaks was highest during the wintering phase of the annual cycle, when 25% (2/8) of UDs were significant. Poultry outbreaks correlated with the wintering distribution of bar-headed geese occurred in India, Bangladesh, Nepal and Lhasa. Spring migration and breeding were each associated with poultry outbreaks for only one of the six (16.7%) UDs. In terms of wild bird outbreaks, only one of the six UDs was significantly associated during the spring migration. Wild bird outbreaks coinciding with the spring migration occurred in Qinghai Lake.

**Table 1:** Association of biweekly utilization distributions (UD) of bar-headed geese and ruddy shelduck with HPAI H5N1 outbreaks in the Central Asian Flyway.

		Poultry outbreaks					Wild bird outbreaks			
		Bar-headed goose		Ruddy shelduck			Bar-headed goose		Ruddy shelduck	
First date	Poultry outbreaks	Proportion associated with UD	p-value	Proportion associated with UD	p-value	Wild bird outbreaks	Proportion associated with UD	p-value	Proportion associated with UD	p-value
25 Nov	12	0.100		0.917	**<0.001**	0				
9 Dec	18	0.000	0.916	0.667	**<0.001**	0				
23 Dec	14	0.455	**0.007**	0.692	**0.001**	0				
8 Jan	39	0.862	**<0.001**	0.564	**<0.001**	0				
22 Jan	74	0.154	0.062	0.521	**<0.001**	0				
5 Feb	69			0.294	**0.050**	0				
19 Feb	54			0.294	**0.037**	0				
4 Mar	77	0.146	0.152	0.453	**<0.001**	0				
18 Mar	101	0.281	**0.012**	0.455	**0.001**	0				
1 Apr	41			0.390	0.172	0				
15 Apr	8	0.000	0.463	0.000	0.866	2	0.500	0.340	1.000	**0.049**
29 Apr	8	0.333	0.276	0.000	0.831	3	1.000	**0.030**	1.000	**0.040**
13 May	9	0.500	0.103	0.000	0.774	6	0.600	0.095	0.400	0.169
27 May	6	0.400	0.335	0.667	**0.005**	2	0.500	0.414	1.000	0.141
10 Jun	7	0.600	**0.040**	0.000	0.595	0				
24 Jun	1			0.000	0.126	0				
8 Jul	2	0.000	0.324	0.000	0.245	0				
22 Jul	1			0.000	0.152	2	0.500	0.283		
5 Aug	0					0				
19 Aug	1					0				
2 Sep	0					0				
16 Sep	3	0.000	0.469	0.000	0.293	0				
30 Sep	0					0				
14 Oct	2	0.000	0.342	0.000	0.206	0				
28 Oct	1	0.000	0.180	0.000	0.117	0				
11 Nov	9	0.000	0.667	0.000	0.537	0				

Strength of association was tested with Fisher's Exact tests and significant p-values (≤0.05) appear in bold.

By comparison, 46.2% (12/26) of the ruddy shelduck UDs were associated with HPAI H5N1 outbreaks (Fig. 3b). Association with poultry outbreaks was highest during wintering when 100% (7/7) of UDs were significant. Poultry outbreaks correlated with the wintering distribution of ruddy shelduck occurred in Myanmar, India and Bangladesh. Spring migration was also associated with poultry outbreaks (but to a lesser degree (42.9%; 3/7). In terms of wild bird outbreaks, only spring migration was significantly associated for 28.6% (2/7) of UDs. Wild bird outbreaks coinciding with the spring migration occurred in Qinghai Lake and Mongolia.

### Phylogeographic relationships of HPAI H5N1 along the Central Asian Flyway

**Figure 4:**
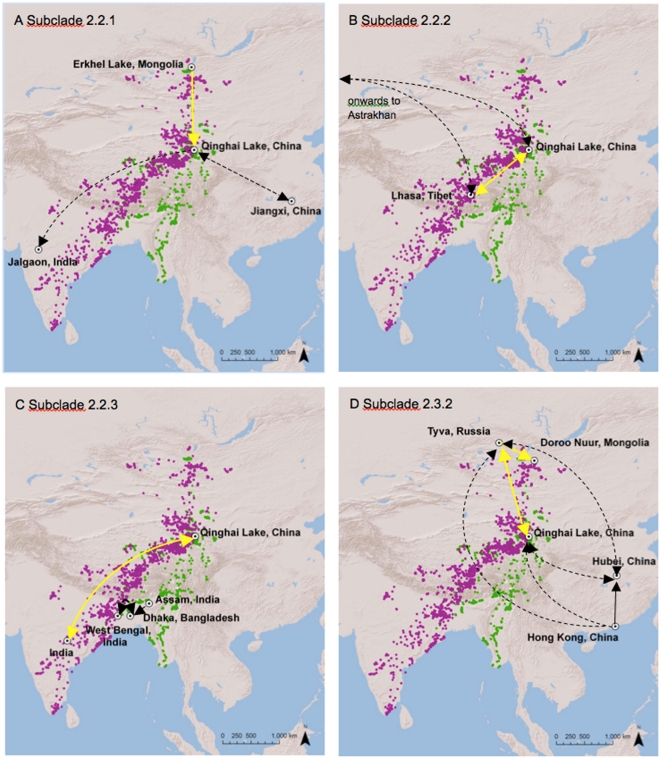
Phylogeographic relationships of HPAI H5N1 in relation to bar-headed geese (purple) and ruddy shelduck (green) in the Central Asian Flyway, 2005–2010. Four subclades characterized the evolution of HPAI H5N1 in wild birds and poultry during this period (a) 2.2.1, (b) 2.2.2, (c) 2.2.3 and (d) 2.3.2. The hypothesized mode of virus movement includes; wild birds (yellow), poultry (black) or inconclusive (dashed).

Phylogeographic mapping of wild bird and poultry HPAI H5N1 isolates identified 6 clades overlapping with the Central Asian Flyway (see Fig. S3). Clade ‘A’ (n = 12 sequences) corresponded with WHO subclade 2.2.1 and revealed a southward pathway, originating in Mongolia and emerging at Qinghai Lake (Fig. 4a). Viruses belonging to clade ‘C’ (n = 16 sequences) also corresponded with WHO subclade 2.2.1 and showed a southward trajectory from Qinghai Lake to Jalgaon, India and a bidirectional pathway between Qinghai Lake and Jiangxi, China (Fig. 4a). Clade ‘B’ (n = 7 isolates) corresponded with WHO subclade 2.2.2 and showed a bidirectional pathway of evolution between Lhasa, Tibet, Qinghai Lake and Astrakhan, Russia (Fig. 4b). Clade ‘E’ (n = 14 sequences) corresponded with WHO subclade 2.2.3 and showed a bidirectional pathway of evolution between Qinghai Lake and Peninsular India. Viruses belonging to clade ‘D’ (n = 55 sequences) also corresponded with WHO subclade 2.2.3 and revealed numerous patterns of movement in South Asia with a primarily northeastward trajectory (Fig. 4c). Viruses were exported 1) from Assam, India into Bangladesh and 2) bidirectionally between West Bengal, India and Bangladesh (Fig. 4c). Viruses belonging to clade ‘F’ (n = 24 sequences) corresponded with WHO subclade 2.3.2 (Fig. 4d). These viruses were exported northwards to Tyva, Russia from Qinghai Lake. Hong Kong was also a source of viruses to Tyva, while Hubei showed bidirectional movement of viruses with Tyva. Viruses also moved southeasterly from Tyva into Mongolia. Qinghai Lake was a sink for viruses originating in Hong Kong and showed bidirectional movement with Hubei, China.

## Discussion

Concurrent investigation of wild bird movements and phylogeographic mapping of HPAI H5N1 links two previously disparate approaches, that combined may provide novel insights into the role of wild birds in HPAI H5N1 dispersal. Our satellite telemetry analysis revealed spatio-temporal overlap between wintering populations of ruddy shelduck (100%) and to a lesser degree, bar-headed geese (25%), with HPAI H5N1 poultry outbreaks in South Asia. The two species showed differing association with poultry outbreaks owing to their relatively distinct wintering distributions. The wintering distribution of bar-headed geese was centered in northern India and Lhasa; however, the ruddy shelduck distribution spanned the Bay of Bengal, coinciding with foci of HPAI H5N1 activity in poultry. This region has been identified as a ‘hotspot’ for outbreaks because of the prevalence of backyard farms that keep mixed flocks of indigenous chickens and ducks, coupled with lack of vaccination [24], [46] that is wide-spread in Bangladesh [47].

Biosecurity of backyard poultry farms in South and Southeast Asia is typically minimal or non-existent, creating opportunities for wild and domestic birds to intermingle at local wetlands [24]. The daily practice of herding domestic ducks into local wetlands and post-harvested rice fields to forage may promote inter-sectoral transmission of the virus, facilitating infection of wild migratory birds from the domestic reservoir. The winter is also characterized by intensive production, increased trade and cold stress in poultry that may contribute to increased susceptibility and shedding duration of domestic hosts. Poultry outbreaks showed strong winter seasonality, peaking between February and March. During this period wild waterfowl are in early hyperphagia, a state of increased foraging to build up body reserves in preparation for long-distance migration [48]. As a consequence, ruddy shelduck and bar-headed geese may be at heightened risk of water-borne transmission when HPAI H5N1 shedding is peaking in poultry.

Outbreaks of HPAI H5N1 in the Central Asian Flyway showed a unique temporal signature with outbreaks in poultry immediately preceding outbreaks in wild birds (see Fig. 1 & 2). This pattern suggests that wild bird outbreaks are likely the result of spillover from a poultry reservoir. This scenario is consistent with evidence from clinical studies indicating that wild birds cannot perpetuate the virus indefinitely [20], [21], [49] and is supported by absence of HPAI H5N1 during sampling of more than 750,000 healthy wild birds as part of global surveillance efforts [50]. Bar-headed geese breeding in Mongolia showed 0.114 probability of moving from wintering grounds in India to Lhasa within an infection-relevant time frame during the spring migration. As revealed by our previous satellite telemetry study, bar-headed geese and ruddy shelduck achieved their fastest rates of travel in the Central Asian Flyway during the spring and autumn migration, showing a maximum potential to disperse the virus during this period [14].

Outbreaks in wild birds were spatially distinct from poultry outbreaks and occurred at stopover or breeding sites north of the Himalayan mountain range: Lhasa in the Tibetan Autonomous Region, Qinghai Lake in Qinghai Province, and Erkhel Lake in Mongolia. With the exception of the Lhasa region, wild bird outbreaks occurred in high altitude regions where poultry production is unsustainable. A prime example is Mongolia, where pastoralism of native sheep, yak and cows is the mainstay of the economy, and poultry production is a small industry restricted to Ulanbaaatar [51]. Similarly, we found that Qinghai Lake was absent of poultry production or captive wild bird colonies during our repeated visits from 2006–2010 [26]. However, we can confirm that a wildlife rescue center with bar-headed geese exists at the reserve and likely did during 2003–2005, raising the possibility that migratory birds did not introduce the virus that caused the large-scale outbreak in 2005 [52].

While trade of both poultry and wild birds are viewed as primarily responsible for the current global distribution of HPAI H5N1 [16], [29], [53] our findings demonstrate that conditions between 2005 and 2010 were conducive to dispersal of HPAI H5N1 with the migration of bar-headed geese and ruddy shelduck. Other notable cases of potential spread of HPAI H5N1 by migratory birds occurred during a sudden cold spell in Europe during 2005–2006. This event was driven by a climate anomaly [22]. The recurring spatio-temporal pattern of wild bird outbreaks in the Central Asian Flyway, sets it apart as unique example of HPAI H5N1 infection in wild birds perpetuated by spillover from poultry in South and Southeast Asia. However, host movement conducive to long-distance dispersal is limited to the narrow window of spring migration.

Bar-headed geese moving through Lhasa have been proposed as the source of HPAI H5N1 at Qinghai Lake during the large-scale 2005 outbreak [26]. Many bar-headed geese that wintered in South Asia staged in the Lhasa or Xigazê Prefectures of the Tibetan Autonomous Region after crossing the Himalaya. Lhasa also proved to be an important wintering ground for bar-headed geese and surveys indicated at least 25% of the global population now winters in this region [54], [55]. Some of the ruddy shelduck also staged in Lhasa; however, their primary pathway was 700 km east of Lhasa. The concentration of bar-headed geese at this migratory bottleneck provides conditions for the sustained transmission of HPAI H5N1 despite limited persistence in the environment [56]. Recent studies have highlighted poultry and captive bar-headed goose farms in Lhasa as a possible source of infection for wintering birds [26], [57].

Qinghai Lake was identified as the major site of convergence between the two waterfowl species. Over 150,000 migratory birds use Qinghai Lake each year as a breeding or staging area [58] with implications for interspecific transmission of HPAI H5N1. The significance of Qinghai Lake as an epicenter for the virus is reflected in the emergence of clade 2.2, the first lineage to infect wildlife including the bar-headed goose and other migratory species in the Central Asian Flyway [59]. The comingling of taxa including ducks, geese, gulls and cormorants at Qinghai Lake may create persistent infection cycles across multiple species [60], [61]. Wild bird outbreaks peaked in May with few cases reported after the spring migration. While the original outbreak at Qinghai Lake in 2005 was sustained from mid-May to late June [18], an outbreak of this scale and duration has not been observed since. Prolonged outbreaks of HPAI H5N1 among waterfowl are rare and as indicated by global surveillance data, are largely restricted to the Central Asian Flyway.

For both bar-headed geese and ruddy shelduck, birds migrating over longer distances flew more rapidly compared to birds with wintering and breeding grounds close together. Ruddy shelduck migrating from the Bay of Bengal to Mongolia were more likely to arrive at Qinghai Lake within an infection-relevant time frame (0.693) compared to those migrating only to Qinghai Lake (0.256). Both species achieved their highest potential to disperse the virus between Qinghai Lake and Mongolia, implying a high risk of virus transmission to Mongolia after an outbreak at Qinghai Lake [12]. Ruddy shelduck showed a higher probability of completing movements within an infection-relevant timeframe (0.708) during this leg of migration compared to bar-headed geese (0.282). This is in contrast with the localized distribution of ruddy shelduck marked in Kazakhstan [62], a reminder that geographically distinct populations have different migration ecology despite both occurring within the Central Asian Flyway. In addition, ruddy shelduck arrived at Qinghai Lake and Mongolia earlier than bar-headed geese and may be responsible for northward advance of HPAI H5N1 into Mongolia.

Our hypothesis that the spring migration of wild birds corresponds with the northward movement of virus was in contrast to the spatial pattern of viral evolution indicated by phylogeographic mapping. Of the four subclades overlapping with the Central Asian Flyway, two subclades moved in a strictly southern direction. Subclade 2.2.1, the first lineage to emerge in wild birds after 2005 [63] dispersed from Mongolia to Qinghai Lake and Qinghai Lake to India. Similarly, subclade 2.3.2 spread from Tyva to Mongolia. Clades 2.2.2 and 2.2.3 were largely bidirectional in their movement, suggesting a complex picture of gene flow in this region. Consequently, phylogeographic mapping suggested a primarily southward movement of the virus along the Central Asian Flyway, coinciding with the autumn migration of wild birds.

Interestingly, no HPAI H5N1 outbreaks involving wild birds have been reported in the Central Asian Flyway during the autumn migration. The possibility exists that HPAI H5N1-infected wild birds were able to migrate without succumbing to clinical disease. For instance, prior exposure to the virus during the spring transmission period may be responsible for inducing immunity among autumn migrants. The small proportion of juvenile birds introduced into the population after June may have been protected by ‘flock immunity’ preventing the virus from gaining a foothold and spreading to susceptible individuals. Sakoda *et al.* [64] documented HPAI H5N1 from dead and moribund spring migrants that had arrived at breeding grounds in Mongolia compared to fall migrants in which the virus was absent. Further immunological studies of wild birds that assess rates of seroconversion during the annual cycle, similar to studies of LPAI [65], are needed to clarify whether build up of antibodies is responsible for the absence of HPAI H5N1 outbreaks during the autumn.

The movement of HPAI H5N1 to South Asia due to the southern migration of wild birds has been proposed by virological studies [30], [66]. However, these investigations have not reconciled how birds become infected when HPAI H5N1 outbreaks in the Central Asian Flyway peak during May and autumn migration occurs three months later. This scenario involves infection of birds from one of three possible sources; 1) spillover from a poultry reservoir in southern Siberia, or 2) environmental persistence of virus in wetlands during the breeding and molt seasons in the Palearctic, or 3) virus persistence through infection of multiple wildlife hosts (including mammals such as pikas, *Ochotona curzoniae* [61]) during the breeding and molt seasons in the Palearctic with no apparent morbidity or mortality. These areas represent knowledge gaps in our understanding of how HPAI H5N1 behaves during the northern hemispheric summer.

The mismatch between satellite telemetry-revealed movements and phylogeographic mapping may highlight a potential bias implicit with using publicly available sequences of HPAI H5N1. Phylogeographic mapping hinges on the quality of sequence information made available from independently collected datasets, and ultimately, data gaps exist. The ideal experimental design for viral data collection would involve representative sampling of all avian hosts in space and time, but this is not always feasible. For example, in rural East Asia where wetlands are inaccessible by major roads, wild bird outbreaks may go undetected in comparison to populated urban centers where poultry outbreaks typically occur. More complete surveillance of HPAI H5N1 through rural East Asia is critical to improving our understanding of how migratory birds contribute to the spread and evolution of HPAI H5N1, relative to poultry trade.

The bidirectional movement of subclade 2.2.2 between Lhasa and Qinghai corresponded with the primary migration of bar-headed geese. Our findings suggest that the seasonal movement of bar-headed geese and other waterfowl species that follow this migration route could have assisted with the circulation of subclade 2.2.2 in China. This is consistent with virological evidence that suggests bar-headed goose isolates from this outbreak share PB2 genes common to HPAI H5N1 circulating in live bird markets in Tibet [67]. The potential to disperse the virus along this migration corridor was only demonstrated by one bar-headed goose (#82079 in 6 days). The majority of bar-headed geese did not achieve rates of travel needed to transport the virus in a single, uninterrupted migration leg, implying that relay transmission between migrants at successive stopovers is a more likely mode of transmission.

The movement of subclade 2.2.3 from Central Indian states into West Bengal, Assam and finally Bangladesh reflects the accepted trajectory of the virus in South Asia [68]. This pathway corresponded with the spring migration of bar-headed geese prior to arriving in Lhasa, however this leg is typically undertaken in long, uninterrupted flight that may preclude opportunities to disperse the virus between local wetlands. Illegal trade of poultry between neighboring states in India and neighboring countries such as Bangladesh may be responsible for the cycle of transmission [68], [69]. Ruddy shelduck winter in proximity to this region, but lack of phylogeographic connectivity between the Bay of Bengal and East Asia implies that this species has not been an agent in the spread of HPAI H5N1. No outbreaks in migratory birds have ever been confirmed in South Asia and wild bird surveillance in this region has not yielded positive results for the virus.

The most recent and widespread lineage to emerge in migratory birds, subclade 2.3.2, demonstrated a northwesterly trajectory from Hong Kong and Hubei in eastern China to Tyva, Russia via Qinghai Lake. The advance of these viruses from the East Asian Flyway to the Central Asian Flyway appeared to be only partly related to migratory birds. For example, the movement of viruses originating in Hong Kong and Hubei were directly connected with Tyva, a migratory route not supported by movement data from a large sample of satellite-marked waterfowl [70]. Phylogenetic studies confirm that viruses belonging to subclade 2.3.2 originated in poultry from Vietnam and southern China in 2004 and advanced northwards along poultry trading routes [63], [71].

The role of other waterbird species such as the great-crested grebe (*Podiceps cristatus*), tufted duck (*Aythya fuligula*), whooper swan (*Cygnus cygnus*) and black-headed gull (*Chroicocephalus ridibundus*) may be key to the dissemination of the now widespread subclade 2.3.2. These species have breeding grounds extending to Siberia and may be responsible for introduction of subclade 2.3.2 into Tyva via Mongolia or Qinghai Lake. Great-crested grebes and little grebes (*Tachybaptus ruficollis*) are understudied as a host species for HPAI H5N1, particularly in view of their role in the large-scale outbreak in western Siberia in July 2005 [72] and in June 2006 [73]. Further investigation of these diving birds is needed to better understand their specific migration routes and pathobiology in response to HPAI H5N1 infection.

In conclusion, previous studies that have mapped the phylogenetic relationships of HPAI H5N1 have relied on wild bird census information to estimate generalized patterns of migration [29], [30]. This has provided insights into how wild birds have contributed to the trajectory of the virus at regional or global scales. However, our findings represent a significant advance by using high resolution, satellite telemetry data to characterize the migration strategies of the bar-headed goose and ruddy shelduck, two migratory hosts for HPAI H5N1 in the Central Asia Flyway. Both species emerged as potential agents for the movement of subclade 2.2.1 between Qinghai Lake and Mongolia in our phylogeographic analyses, while the movement of subclade 2.2.2 between Lhasa and Qinghai Lake specifically implicated the bar-headed goose.

Spatio-temporal analysis of outbreaks revealed that the ruddy shelduck was at high risk of exposure to HPAI H5N1 outbreaks in South and Southeast Asia, given their wintering distribution encompassing Bangladesh, Myanmar and India. This species also had a greater potential to hypothetically disperse virus in a single, uninterrupted migration event between the Bay of Bengal and Qinghai or Mongolia. In contrast, the bar-headed goose is most likely to come into contact with HPAI H5N1 at Lhasa, a migratory bottleneck that supports poultry and captive facilities of wild birds, although exposure farther south in India or Bangladesh and northern migration is plausible [24]. Relay transmission involving multiple infected individuals is a more probable mode of transmission for the bar-headed goose in view of their low potential to spread the virus over long distances within the asymptomatic period.

Our results indicate a need for targeted surveillance in South Asia and Lhasa to identify agricultural practices or environmental conditions promoting transmission between migratory birds and poultry. In this region, the intensification of chicken and poultry production in the last 30 years has become entangled with the more traditional practice of rice growing, providing favorable conditions for the transmission of HPAI H5N1 [4]. Protection of wetland habitat throughout Asia that reduces the poultry interface may help to limit cross-infection [74]. Enhanced biosecurity and conservation measures may curb infection of wild birds prior to their arrival at Qinghai Lake and offer the best means of minimizing export of the virus globally via migratory birds with distributions that overlap in the Central Asian Flyway.

## Supporting Information

Figure S1 **Pattern of HPAI H5N1 outbreaks in the Central Asian Flyway, 2005–2010.** Outbreak data based on EMPRES-i global animal health information system (FAO). The wild bird species and the number of individuals (where possible) involved is shown.
(DOC)

Figure S2 **Spatial extent of HPAI H5N1 outbreaks determined by minimum convex polygon to assess migration corridor of wild birds and utilization distributions encompassing two week periods.**
(DOC)

Figure S3 **Phylogenetic tree of HPAI H5N1 (3459 isolates) based on the hemagglutinin gene.** Clades overlapping within the Central Asian Flyway (A,B,C,D,E and F) are highlighted (red). Clades are named according to the World Health Organization system of nomenclature.
(PDF)

Table S1 **Potential for long-distance movement of HPAI H5N1 during the spring migration.** Probabilities express the number of days an individual flew a cumulative distance of >500 km, >1000 km and >1200 km within the 7 day asymptomatic period of infection for (a) bar-headed geese and (b) ruddy shelduck.
(DOCX)
