# Supplementary material for: Eco-Virological Approach for Assessing the Role of Wild Birds in the Spread of Avian Influenza H5N1 along the Central Asian Flyway
Source: PLoS One. 2012 Feb 7;7(2):e30636. doi: 10.1371/journal.pone.0030636 (PMC3274535; doi:10.1371/journal.pone.0030636)
Supplement: Figure S1 — Pattern of HPAI H5N1 outbreaks in the Central Asian Flyway, 2005–2010. Outbreak data based on EMPRES-i global animal health information system (FAO). The wild bird species and the number of individuals (where possible) involved is shown. (DOC) [file pone.0030636.s001.doc]

**Figure S1. Pattern of highly pathogenic H5N1outbreaks in the Central Asian Flyway between 2005 and 2010 based on EMPRES-i global animal health information system (FAO).** The wild bird species and the number of individuals (where possible) involved is shown.


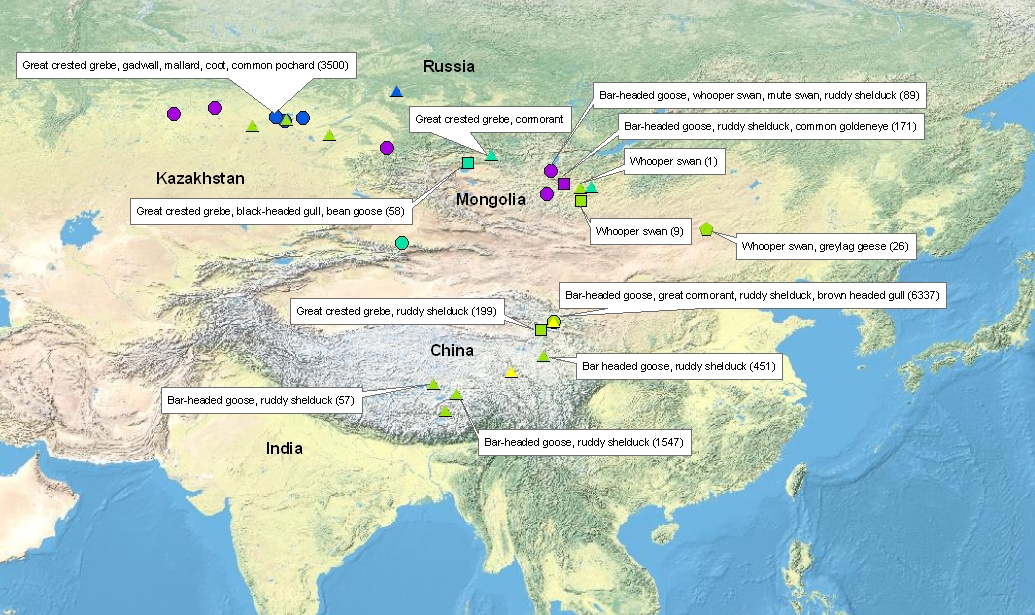


2005

2006

2009

2010

April

May

June

July

August
