# Supplementary figures and images for: Eco-Virological Approach for Assessing the Role of Wild Birds in the Spread of Avian Influenza H5N1 along the Central Asian Flyway
Source: PLoS One. 2012 Feb 7;7(2):e30636. doi: 10.1371/journal.pone.0030636 (PMC3274535; doi:10.1371/journal.pone.0030636)

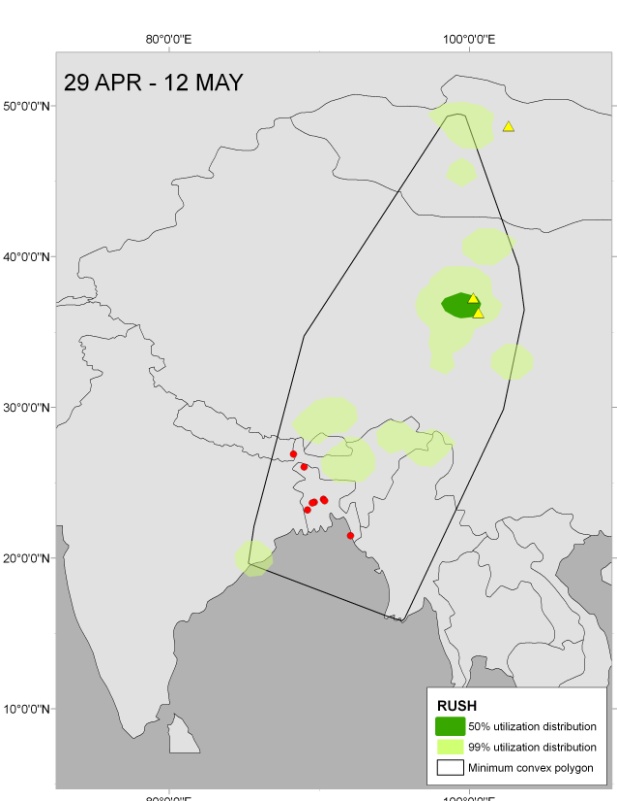

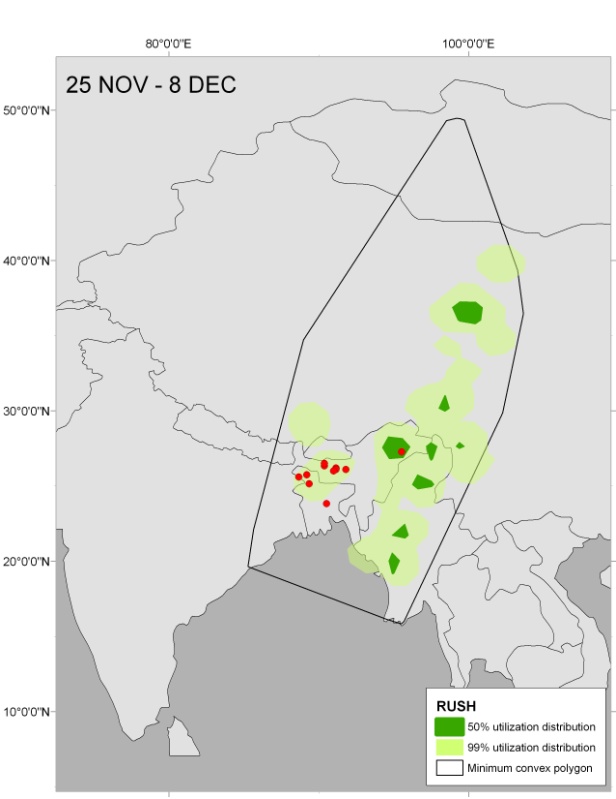

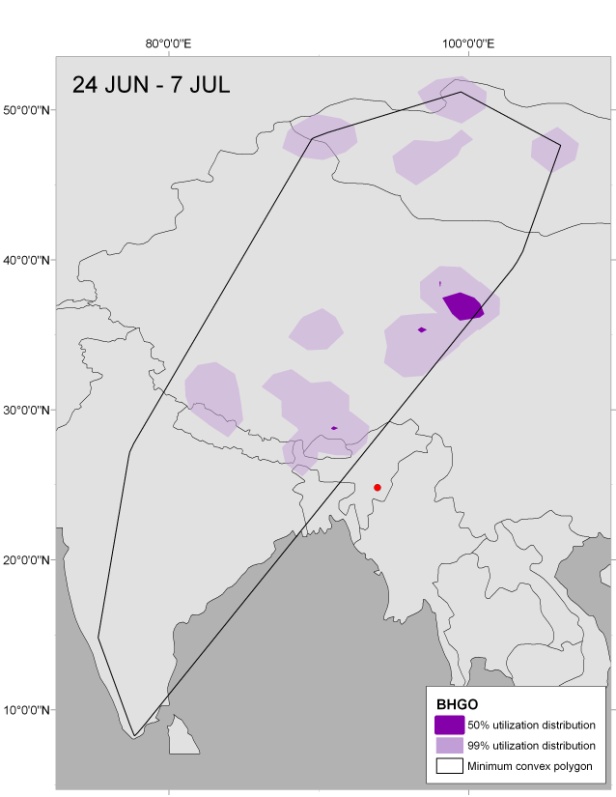

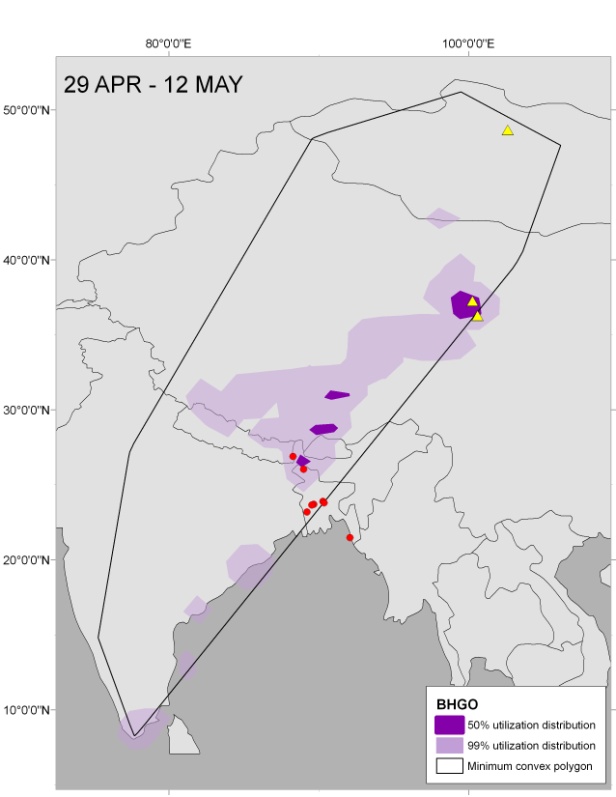

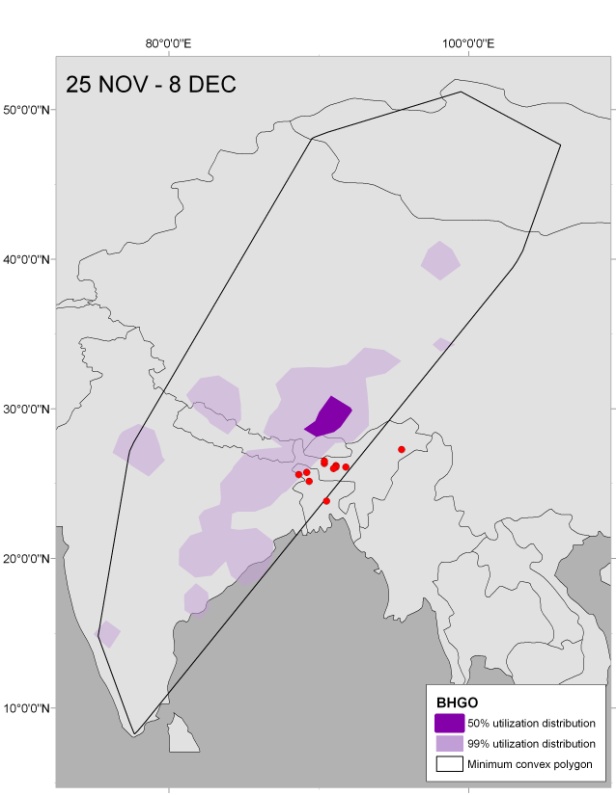


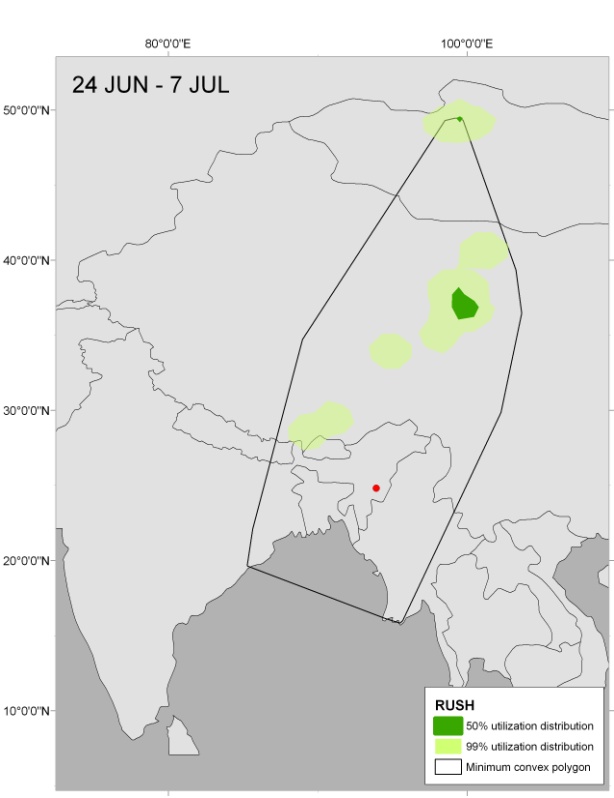

Supplement: Figure S2 — Spatial extent of HPAI H5N1 outbreaks determined by minimum convex polygon to assess migration corridor of wild birds and utilization distributions encompassing two week periods. (DOC) [file pone.0030636.s002.doc]

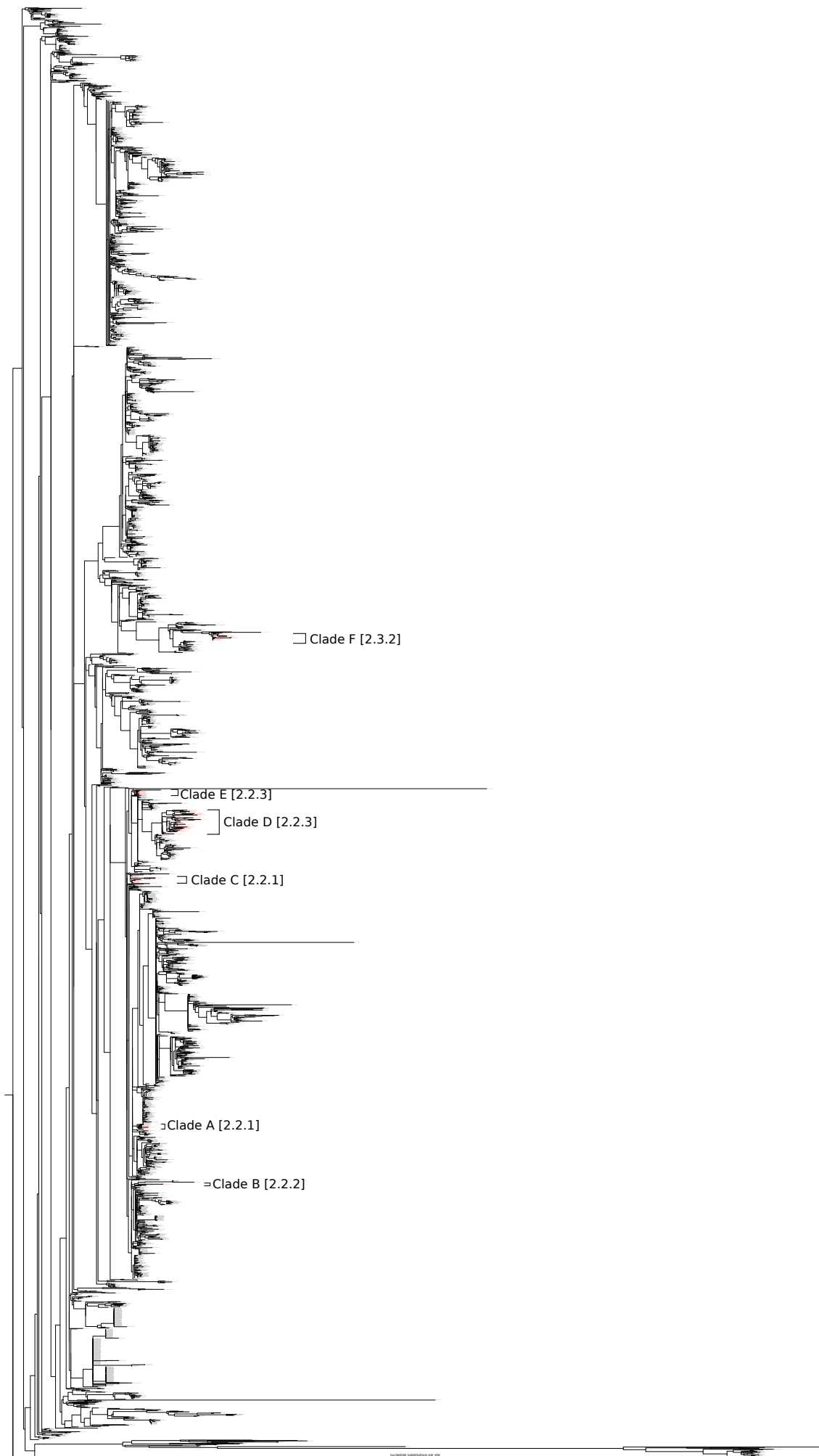

Supplement: Figure S3 — Phylogenetic tree of HPAI H5N1 (3459 isolates) based on the hemagglutinin gene. Clades overlapping within the Central Asian Flyway (A,B,C,D,E and F) are highlighted (red). Clades are named according to the World Health Organization system of nomenclature. (PDF) [file pone.0030636.s003.pdf]
