# Supplementary material for: Eco-Virological Approach for Assessing the Role of Wild Birds in the Spread of Avian Influenza H5N1 along the Central Asian Flyway
Source: PLoS One. 2012 Feb 7;7(2):e30636. doi: 10.1371/journal.pone.0030636 (PMC3274535; doi:10.1371/journal.pone.0030636)
Supplement: Table S1 — Potential for long-distance movement of HPAI H5N1 during the spring migration. Probabilities express the number of days an individual flew a cumulative distance of >500 km, >1000 km and >1200 km within the 7 day asymptomatic period of infection for (a) bar-headed geese and (b) ruddy shelduck. (DOCX) [file pone.0030636.s004.docx]

**Table 1. Potential for long-distance movement of HPAI H5N1 during the spring migration.** Probabilities express the number of days an individual flew a cumulative distance of >500km, >1000km and >1200km within the 7 day asymptomatic period of infection for (a) bar-headed geese and (b) ruddy shelduck.

| **a) Bar-headed goose** | |  |  | Migration leg | | | | | | | | | | | | | | |
| --- | --- | --- | --- | --- | --- | --- | --- | --- | --- | --- | --- | --- | --- | --- | --- | --- | --- | --- |
|  | |  |  | Gangetic Plain to Tibet | | |  | Peninsular India to Lhasa | | |  | Lhasa to Qinghai | | |  | Qinghai to Mongolia | | |
| Route | Individual |  | Days | 500km | 1000km | 1200km | Days | 500km | 1000km | 1200km | Days | 500km | 1000km | 1200km | Days | 500km | 1000km | 1200km |
| Gangetic Plain to Tibet |  |  |  |  |  |  |  |  |  |  |  |  |  |  |  |  |  |  |
|  | BH05_55904 | | 36 | 0.056 | 0.000 | 0.000 |  |  |  |  |  |  |  |  |  |  |  |  |
|  | BH05_55905 | | 42 | 0.048 | 0.024 | 0.000 |  |  |  |  |  |  |  |  |  |  |  |  |
|  | BH05_55906 | | 4 | 1.000 | 0.000 | 0.000 |  |  |  |  |  |  |  |  |  |  |  |  |
|  | BH05_55907 | | 4 | 0.750 | 0.000 | 0.000 |  |  |  |  |  |  |  |  |  |  |  |  |
|  | *Average* |  | *22* | *0.463* | *0.006* | *0.000* |  |  |  |  |  |  |  |  |  |  |  |  |
| Peninsular India to Lhasa |  |  |  |  |  |  |  |  |  |  |  |  |  |  |  |  |  |  |
|  | BH08_85716 | |  |  |  |  | 40 | 0.150 | 0.000 | 0.000 |  |  |  |  |  |  |  |  |
|  | BH08_85786 | |  |  |  |  | 74 | 0.095 | 0.000 | 0.000 |  |  |  |  |  |  |  |  |
|  | BH08_85794 | |  |  |  |  | 67 | 0.090 | 0.000 | 0.000 |  |  |  |  |  |  |  |  |
|  | *Average* |  |  |  |  |  | *60* | *0.112* | *0.000* | *0.000* |  |  |  |  |  |  |  |  |
| Lhasa to Qinghai |  |  |  |  |  |  |  |  |  |  |  |  |  |  |  |  |  |  |
|  | BH07_67582 | |  |  |  |  |  |  |  |  | 17 | 0.353 | 0.000 | 0.000 |  |  |  |  |
|  | BH07_67695 | |  |  |  |  |  |  |  |  | 18 | 0.389 | 0.000 | 0.000 |  |  |  |  |
|  | BH07_74901 | |  |  |  |  |  |  |  |  | 48 | 0.000 | 0.000 | 0.000 |  |  |  |  |
|  | BH07_74902 | |  |  |  |  |  |  |  |  | 34 | 0.353 | 0.000 | 0.000 |  |  |  |  |
|  | BH08_82079 | |  |  |  |  |  |  |  |  | 6 | 0.833 | 0.667 | 0.000 |  |  |  |  |
|  | BH08_82080 | |  |  |  |  |  |  |  |  | 15 | 0.467 | 0.000 | 0.000 |  |  |  |  |
|  | BH08_82082 | |  |  |  |  |  |  |  |  | 38 | 0.184 | 0.000 | 0.000 |  |  |  |  |
|  | BH08_82084 | |  |  |  |  |  |  |  |  | 48 | 0.104 | 0.000 | 0.000 |  |  |  |  |
|  | BH08_82086 | |  |  |  |  |  |  |  |  | 28 | 0.250 | 0.000 | 0.000 |  |  |  |  |
|  | *Average* |  |  |  |  |  |  |  |  |  | *28* | *0.326* | *0.074* | *0.000* |  |  |  |  |
| Peninsular India to Qinghai |  |  |  |  |  |  |  |  |  |  |  |  |  |  |  |  |  |  |
|  | BH08_85715 | |  |  |  |  | 50 | 0.280 | 0.080 | 0.020 | 11 | 0.182 | 0.000 | 0.000 |  |  |  |  |
|  | BH08_85778 | |  |  |  |  | 56 | 0.339 | 0.071 | 0.054 | 16 | 0.188 | 0.063 | 0.000 |  |  |  |  |
|  | BH08_85785 | |  |  |  |  | 48 | 0.271 | 0.146 | 0.000 | 14 | 0.071 | 0.000 | 0.000 |  |  |  |  |
|  | BH08_82081 | |  |  |  |  | 34 | 0.256 | 0.154 | 0.000 | 10 | 0.700 | 0.300 | 0.000 |  |  |  |  |
|  | *Average* |  |  |  |  |  | *47* | *0.287* | *0.113* | *0.019* | *13* | *0.285* | *0.910* | *0.000* |  |  |  |  |
| Peninsular India to Mongolia |  |  |  |  |  |  |  |  |  |  |  |  |  |  |  |  |  |  |
|  | BH08_85718 | |  |  |  |  | 40 | 0.500 | 0.125 | 0.100 | 15 | 0.467 | 0.267 | 0.000 | 11 | 0.727 | 0.091 | 0.000 |
|  | BH08_85722 | |  |  |  |  | 30 | 0.233 | 0.200 | 0.167 | 15 | 0.400 | 0.333 | 0.133 | 6 | 0.333 | 0.333 | 0.333 |
|  | BH08_85777 | |  |  |  |  | 37 | 0.514 | 0.189 | 0.162 | 7 | 0.857 | 0.429 | 0.143 | 7 | 0.857 | 0.857 | 0.714 |
|  | BH08_85784 | |  |  |  |  | 61 | 0.164 | 0.000 | 0.000 | 8 | 0.375 | 0.375 | 0.000 | 11 | 0.636 | 0.364 | 0.364 |
|  | BH08_85792 | |  |  |  |  | 43 | 0.140 | 0.140 | 0.140 | 22 | 0.227 | 0.000 | 0.000 | 31 | 0.290 | 0.226 | 0.000 |
|  | *Average* |  |  |  |  |  | *42* | *0.310* | *0.131* | *0.114* | *13* | *0.465* | *0.281* | *0.055* | *13* | *0.569* | *0.374* | *0.282* |
|  |  |  |  |  |  |  |  |  |  |  |  |  |  |  |  |  |  |  |

| **b) Ruddy shelduck** | |  | Migration leg | | | | | | | | | | | | | | |
| --- | --- | --- | --- | --- | --- | --- | --- | --- | --- | --- | --- | --- | --- | --- | --- | --- | --- |
|  | |  | NE India to Lhasa | | |  | Peninsular India to Tibet | | |  | Bay of Bengal to Qinghai | | |  | Qinghai to Mongolia | | |
| Strategy | Individual | Days | 500km | 1000km | 1200km | Days | 500km | 1000km | 1200km | Days | 500km | 1000km | 1200km | Days | 500km | 1000km | 1200km |
| NE India to Lhasa |  |  |  |  |  |  |  |  |  |  |  |  |  |  |  |  |  |
|  | SD09_95381 | 39 | 0.128 | 0.000 | 0.000 |  |  |  |  |  |  |  |  |  |  |  |  |
| Peninsular India to Tibet |  |  |  |  |  |  |  |  |  |  |  |  |  |  |  |  |  |
|  | SD08_74821 |  |  |  |  | 66 | 0.106 | 0.000 | 0.000 |  |  |  |  |  |  |  |  |
| Bay of Bengal to Qinghai |  |  |  |  |  |  |  |  |  |  |  |  |  |  |  |  |  |
|  | SD07_74813_2008 | |  |  |  |  |  |  |  | 4 | 1.000 | 0.500 | 0.000 |  |  |  |  |
|  | SD07_74816_2008 | |  |  |  |  |  |  |  | 22 | 0.091 | 0.000 | 0.000 |  |  |  |  |
|  | SD07_74822_2008 | |  |  |  |  |  |  |  | 46 | 0.152 | 0.000 | 0.000 |  |  |  |  |
|  | SD08_82095_2009 | |  |  |  |  |  |  |  | 5 | 0.100 | 0.400 | 0.000 |  |  |  |  |
|  | SD08_82095_2010 | |  |  |  |  |  |  |  | 11 | 0.546 | 0.273 | 0.000 |  |  |  |  |
|  | SD08_82097_2009 | |  |  |  |  |  |  |  | 4 | 1.000 | 1.000 | 0.750 |  |  |  |  |
|  | SD08_82099_2009 | |  |  |  |  |  |  |  | 4 | 0.750 | 0.500 | 0.000 |  |  |  |  |
|  | SD08_82099_2010 | |  |  |  |  |  |  |  | 5 | 0.600 | 0.600 | 0.600 |  |  |  |  |
|  | SD08_82100_2009 | |  |  |  |  |  |  |  | 9 | 0.667 | 0.667 | 0.667 |  |  |  |  |
|  | SD08_82100_2010 | |  |  |  |  |  |  |  | 14 | 0.286 | 0.286 | 0.286 |  |  |  |  |
|  | SD08_82119_2009 | |  |  |  |  |  |  |  | 6 | 0.667 | 0.667 | 0.000 |  |  |  |  |
|  | SD08_82121_2009 | |  |  |  |  |  |  |  | 22 | 0.500 | 0.227 | 0.227 |  |  |  |  |
|  | SD08_82122_2009 | |  |  |  |  |  |  |  | 6 | 0.333 | 0.000 | 0.000 |  |  |  |  |
|  | SD08_82122_2010 | |  |  |  |  |  |  |  | 5 | 0.400 | 0.400 | 0.400 |  |  |  |  |
|  | SD08_82126_2009 | |  |  |  |  |  |  |  | 4 | 0.750 | 0.000 | 0.000 |  |  |  |  |
|  | SD08_82126_2010 | |  |  |  |  |  |  |  | 5 | 0.800 | 0.800 | 0.200 |  |  |  |  |
|  | SD08_82127_2009 | |  |  |  |  |  |  |  | 5 | 0.800. | 0.600 | 0.600 |  |  |  |  |
|  | SD08_82127_2010 | |  |  |  |  |  |  |  | 8 | 0.875 | 0.875 | 0.875 |  |  |  |  |
|  | *Average* | |  |  |  |  |  |  |  | *10* | *0.623* | *0.433* | *0.256* |  |  |  |  |
| Bay of Bengal to Mongolia |  | |  |  |  |  |  |  |  |  |  |  |  |  |  |  |  |
|  | SD07_74808_2008 | |  |  |  |  |  |  |  | 2 | 1.000 | 1.000 | 1.000 | 4 | 0.750 | 0.500 | 0.500 |
|  | SD07_74810_2008 | |  |  |  |  |  |  |  | 3 | 1.000 | 1.000 | 1.000 | 5 | 1.000 | 1.000 | 1.000 |
|  | SD07_74810_2009 | |  |  |  |  |  |  |  | 16 | 0.438 | 0.438 | 0.438 | 3 | 0.667 | 0.667 | 0.667 |
|  | SD07_74810_2010 | |  |  |  |  |  |  |  | 6 | 1.000 | 1.000 | 0.333 | 3 | 1.000 | 1.000 | 0.667 |
|  | *Average* | |  |  |  |  |  |  |  | *7* | *0.859* | *0.859* | *0.693* | *4* | *0.854* | *0.792* | *0.708* |
|  |  | |  |  |  |  |  |  |  |  |  |  |  |  |  |  |  |
